# Real-World Treatment Patterns, Healthcare Resource Utilization, and Healthcare Costs in the First-Line Treatment of Metastatic Non-Small Cell Lung Cancer in the US

**DOI:** 10.3390/curroncol32030151

**Published:** 2025-03-05

**Authors:** Divyan Chopra, David M. Waterhouse, Ihtisham Sultan, Björn Stollenwerk

**Affiliations:** 1 Amgen, Thousand Oaks, CA 91320, USA; 2 OHC (Oncology Hematology Care), Cincinnati, OH 45242, USA; 3 Amgen (EUROPE) GmbH, 6343 Rotkreuz, Switzerland

**Keywords:** economic burden, immunotherapy, chemotherapy, observational, claims data

## Abstract

This study characterizes real-world treatment patterns and economic and healthcare resource utilization (HCRU) burden associated with first-line (1L) treatment of metastatic non-small cell lung cancer (NSCLC) without actionable alterations in the United States. This retrospective observational study used Optum Clinformatics^®^ data. A total of 15,659 patients with metastatic NSCLC who started 1L treatment between January 2020 and March 2023 were included (52% male; mean age at the start of 1L treatment 71.7 years; 86% Medicare Advantage). The most frequent 1L regimens were immune checkpoint inhibitor (ICI) + platinum-based chemotherapy (PBCT) (47%), PBCT only (26%), and ICI only (20%). The median 1L treatment duration was 4.2 months (range 2.7–6.5) and was shorter with chemotherapy-only regimens. Outpatient visits accounted for the majority of HCRU (mean 6.6 visits per patient per month [PPPM]). Outpatient, inpatient, and emergency department visits were highest for chemotherapy-only regimens. Mean total (all-cause) healthcare costs were $32,215 PPPM and were highest for ICI + chemotherapy ($34,741–38,454 PPPM). Inpatient costs PPPM were highest for PBCT ($4725) and ICI + non-PBCT ($4648). First-line treatment of metastatic NSCLC without actionable alterations imposes a notable HCRU and cost burden, underscoring the need for better treatment options to improve outcomes and reduce economic impact.

## 1. Introduction

Lung cancer is a primary cause of cancer-related death in the United States (US) and globally [1,2]. The majority of lung cancer diagnoses are non-small-cell lung cancer (NSCLC) [3], with most diagnoses occurring at advanced or metastatic stages. Almost half of patients diagnosed with early-stage disease experience disease progression within 5 years [4]. Patients with advanced or metastatic NSCLC demonstrate poor survival despite advances in treatment, with 5-year survival rates of 10–15% [4,5]. Molecular subtypes, such as Kirstin rat sarcoma virus (KRAS) mutations, are known to significantly impact patient-reported outcomes and overall disease burden, emphasizing the need for tailored therapeutic approaches [6].

Patients with advanced/metastatic NSCLC have been historically treated with chemotherapy regimens [7]. However, the development of new systemic anticancer therapies (SACT) such as anti-angiogenic agents, immune checkpoint inhibitors (ICIs) and therapies that target specific alterations (e.g., endothelial growth factor receptor, anaplastic lymphoma kinase, Kristen rat sarcoma (KRAS)) [7,8] have led to a shift in the treatment landscape for NSCLC. Targeted therapies have demonstrated improved survival in patients with actionable alterations and are generally better tolerated than chemo(immuno)therapy [9]. For instance, sotorasib has been shown to improve clinical and patient-reported outcomes compared with docetaxel in patients with KRAS G12C-mutated NSCLC, as demonstrated in the CodeBreaK 200 trial [10,11]. However, 50–60% of patients with advanced NSCLC do not have actionable alterations [8,9], and treatment options for these patients have become increasingly complex. ICIs, which were approved in the US in 2016 for the first-line (1L) treatment of metastatic NSCLC, either as monotherapy or in combination with platinum-based chemotherapy (PBCT), have become established as 1L treatment [12,13] based on survival benefits demonstrated in clinical trials [14]. Despite advances in treatment options, decision-making in the real-world setting remains complex, particularly for patients without actionable alterations. Clinical trial populations often differ from real-world patients in terms of baseline characteristics, comorbidities, and treatment eligibility, which may impact outcomes and treatment choices [15]. It is important to understand the uptake of these treatments in real-world settings and to evaluate the overall economic and resource burden of 1L treatments on healthcare systems.

Although several studies have reported the burden of illness in US clinical practice for patients with metastatic NSCLC [16,17,18,19,20,21,22], most focus on second and later lines of therapy, evaluate targeted treatments or use data that largely precede the widespread adoption of ICIs. The current study aims to address this knowledge gap by evaluating real-world treatment patterns, healthcare resource utilization (HCRU), and healthcare costs, specifically among patients receiving SACTs for the 1L treatment of metastatic NSCLC without actionable alterations in the US. Understanding real-world outcomes can provide valuable insights for clinicians, policymakers, and payers in terms of managing healthcare expenditure and treatment options in the 1L treatment of NSCLC.

## 2. Materials and Methods

This was a retrospective observational cohort study utilizing data from the Optum Deidentified Clinformatics^®^ database. The Optum database includes information on enrollment medical and pharmacy data for persons insured in the US via commercial insurance, as well as Medicare Advantage plans. Enrollment information includes age, sex, region, payer, plan type, and date of plan enrollment. Medical data include dates of service use, diagnosis, procedure information, and financial information. Pharmacy data include type of prescription filled, dates of filling, quantity, number of days’ supply, and financial information.

### 2.1. Study Design and Population

**Figure 1:**
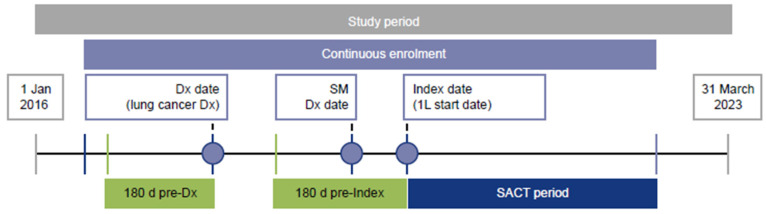
Study design. The SACT period is the time from the index date until the earliest of 30 days after the last cycle of SACT during the LOT, the day before the next LOT or the end of follow-up. 1L, first-line; d, days; Dx, diagnosis; SACT, systemic anticancer treatment; SM, secondary malignancy.

Eligible patients had a diagnosis of lung cancer (date of first diagnosis = Dx date) during the study period (1 January 2016 to 31 March 2023), a diagnosis of metastases on or after the Dx date (secondary malignancy date) and had received 1L SACT for lung cancer following the secondary malignancy date. The initiation of 1L SACT was determined as the index date. The study design is shown in Figure 1. The International Classification of Diseases, 10th Edition, Clinical Modification (ICD-10-CM) code C34 was used for identification of lung cancer diagnosis.

Lines of treatment were determined using an adaptation of a previously published algorithm [23]: the date of first metastatic disease was determined to be the index date. The date of the first SACT received after the index date was the start date of 1L treatment. All SACTs received within the 30-day period beginning with the 1L treatment start date were classified as a part of the 1L regimen. The line of treatment (LOT) advanced when a new drug that was not part of the current regimen was introduced or if there was a treatment gap of at least 60 days. However, temporary pauses or reintroductions of drugs within the same regimen did not change the LOT. Substitutions between similar drugs (e.g., cisplatin and carboplatin, paclitaxel and albumin-bound paclitaxel) or the addition of National Comprehensive Cancer Network (NCCN)-recommended maintenance therapies for metastatic NSCLC did not advance the LOT. LOT is finished at the end of continuous enrollment, the end of the study period, or death.

Patients were also required to initiate 1L SACT on or after 1 January 2020 (to ensure capture of recent data), to be at least 18 years of age as of the index date and to have been continuously enrolled in medical and pharmacy benefits from a minimum of 180 days before the Dx date to a minimum of 30 days after the index date. Exclusion criteria included receiving treatments for actionable mutations (not including ICIs or vascular endothelial growth factor therapies). In order to ensure that patients with NSCLC were captured, those receiving treatments typically administered for small-cell lung cancer (SCLC) before the index date were excluded. Patients receiving SACT for lung cancer and those who had a diagnosis of metastases during the 180-day pre-diagnosis period were also excluded.

### 2.2. Treatment Classification

Treatment regimens for 1L were classified into the following categories: ICI therapy (mono/dual), ICI + PBCT, ICI + non-PBCT, PBCT mono/combination therapies (excluding ICIs), non-PBCT monotherapies, and non-PBCT combination therapies (excluding ICIs), plus any other regimens that were identified during the analysis.

### 2.3. Baseline Characteristics

Demographics assessed on the index date included age, region, sex and payer. Clinical characteristics included types of secondary metastases, comorbidities that are common among lung cancer patients (such as anemia, chronic obstructive pulmonary disease [COPD], chronic kidney disease, chronic liver disease, coronary heart disease, dementia, diabetes, hypertension and cerebrovascular disease), smoking history and National Cancer Institute (NCI) Adapted Charlson Comorbidity Index (NCI-CCI) [24]. Clinical characteristics were assessed during the 180 days up to but excluding the index date.

### 2.4. Study Outcomes

The duration of 1L treatment was defined as the time from the index date to the earliest maximum runout date for all the therapies in 1L, the day before the start date of the next line of treatment, study end date or end of follow-up. For patients who went on to receive second-line (2L) treatment, time to next treatment (TTNT) was estimated as the number of days from the start of 1L treatment to the day before the start of 2L treatment.

All-cause and NSCLC-related HCRU and healthcare costs were assessed during the SACT treatment period, which spanned from the index date to the earliest of 30 days after the maximum runout date for all treatments in 1L regimen, the day before the start of 2L, or the end of the follow-up period. All-cause and NSCLC-related HCRU and healthcare costs were evaluated at the per-patient per-month (PPPM) level and stratified into outpatient (including chemotherapy and ICI costs as these are generally administered in an outpatient setting), inpatient, emergency department (ED) and pharmacy (prescriptions). A claim was considered to be NSCLC-related if it had a diagnosis of lung cancer in any position or if it was for a lung-cancer-related medication.

### 2.5. Analysis

No statistical testing was carried out, and the analyses were presented descriptively. Means (standard deviation [SD] or medians (interquartile range [IQR]) are used for reporting continuous variables, and numbers and percentages are used for the reporting of categorical variables. To account for inflation, healthcare costs were adjusted to 2022 US dollars using the medical component of the Consumer Price Index [25]. To account for censoring, treatment duration was determined using Kaplan–Meier survival analysis.

STROBE (Strengthening the Reporting of Observational Studies in Epidemiology) guidelines for reporting cohort studies were considered.

## 3. Results

### 3.1. Study Population

A total of 15,659 patients met the eligibility criteria (Table 1). Their mean age at the start of 1L treatment was 71.7 (SD 8.6) years; 52% were male, and the majority (86%) of patients were covered by Medicare Advantage (Table 2). The median duration from lung cancer diagnosis to initiation of 1L therapy was 1.5 (IQR 0.9–2.8) months. The mean NCI-CCI score was 2.3 (SD 1.9). The most common comorbidities reported were hypertension (73%), COPD (44%) and coronary heart disease (29%).

### 3.2. Treatment Patterns

**Figure 2:**
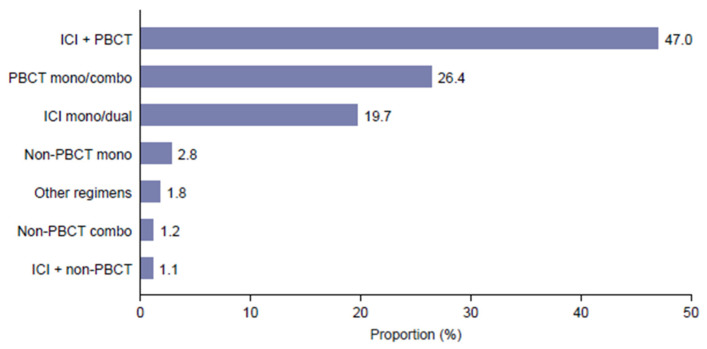
Distribution of first-line treatments. ICI is an immune checkpoint inhibitor; PBCT is a platinum-based chemotherapy.

The most frequent 1L treatment regimens were ICI + PBCT (*n* = 7359; 47.0%), PBCT mono/combo (*n* = 4139; 26.4%) and ICI mono/dual (*n* = 3089; 19.7%) (Figure 2). Additional details on treatment patterns are provided in Supplementary Table S1.

**Figure 3:**
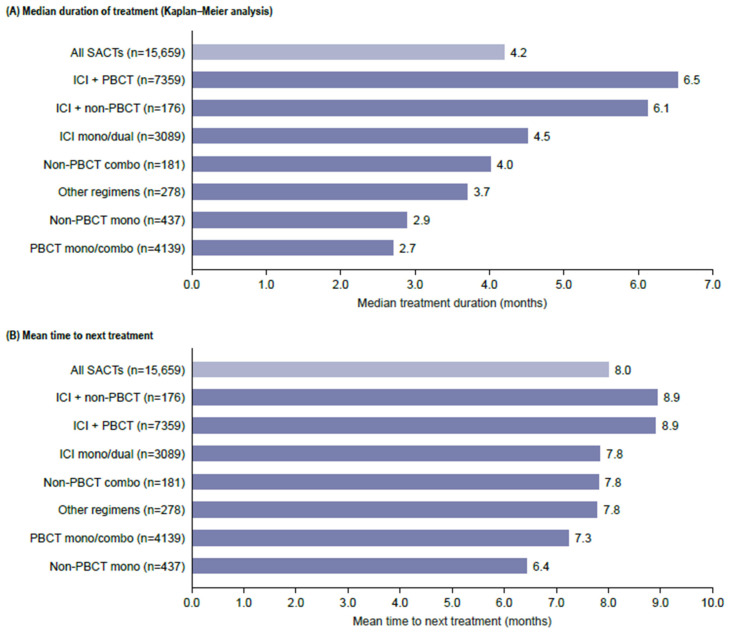
Duration of treatment and time to next treatment. ICI is an immune checkpoint inhibitor; PBCT is a platinum-based chemotherapy; SACTs, systemic anti-cancer treatment.

The mean duration of follow-up was 11.2 (SD 9.2) months. The median duration of 1L treatment (based on Kaplan–Meier analysis) was 4.2 months; the mean TTNT among patients who received 2L treatment was 8.0 months (SD 5.7). The treatment duration and TTNT were the shortest for regiments containing chemotherapy alone (Figure 3).

### 3.3. HCRU

**Figure 4:**
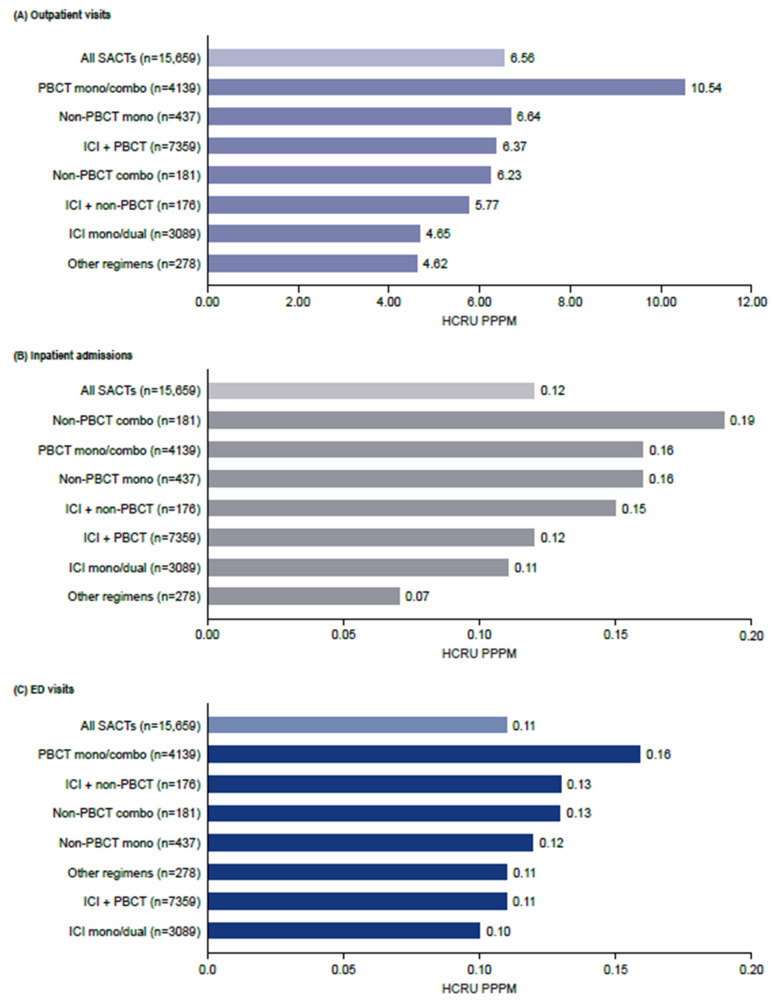
Healthcare resource utilization during the first-line treatment of NSCLC. Combo, combination; HCRU, healthcare resource utilization; ICI, immune checkpoint inhibitor; PBCT, platinum-based chemotherapy; PPPM, per patient per month; SACT, systemic anticancer therapy.

Chemotherapy-only regimens were associated with the greatest HCRU for outpatient, inpatient and ED visits across all treatment categories (Figure 4). Outpatient visits accounted for the majority of all-cause HCRU. The mean number of outpatient visits was 6.6 PPPM and was highest for PBCT mono/combo (10.5 PPPM) and non-PBCT monotherapy (6.6 PPPM) (Figure 4A). Almost three-quarters of outpatient visits (74%) were NSCLC-related (Supplementary Table S1). The mean number of all-cause inpatient admissions was 0.12 PPPM and was highest for non-PBCT combinations (0.19 PPPM), PBCT mono/combination therapies and non-PBCT therapies (both 0.16 PPPM). NSCLC-related inpatient admissions were also highest for PBCT-containing regimens (Figure 4B). The mean number of ED visits was 0.11 PPPM and was highest with PBCT mono/combination therapies (0.16 PPPM) (Figure 4C).

### 3.4. Healthcare Costs

**Figure 5:**
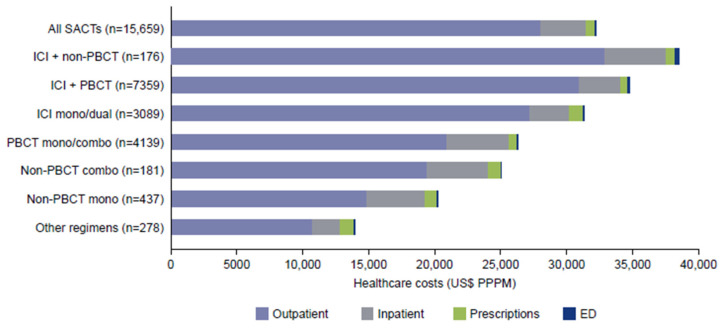
Mean total (all-cause) healthcare costs during first-line treatment. Combo, combination; ED, emergency department; ICI, immune checkpoint inhibitor; PBCT, platinum-based chemotherapy; PPPM, per patient per month; SACT, systemic anticancer therapy.

Figure 5 illustrates the relative contributions of the key cost categories (inpatient, outpatient, ER, prescriptions) to the total cost for each treatment type. Mean total (all-cause) healthcare costs during 1L treatment were $32,215 PPPM (SD 44,597) and were highest for ICI in combination with chemotherapy regimens: $38,454 (SD 55,678) for ICI + non-PBCT, $34,721 (SD 44,191) for ICI + PBCT (all PPPM) (Figure 5).

All-cause healthcare costs were largely incurred in outpatient settings ($28,045 [SD 37,735] PPPM), followed by inpatient costs ($3412 [SD 21,791] PPPM). Outpatient costs (PPPM) were highest for ICI in combination with chemotherapy: $32,913 (SD 50,790) for ICI + non-PBCT, followed by $30,888 (SD 37,409) for ICI + PBCT. Inpatient costs were highest for PBCT mono/combination therapy ($4725 [SD 22,874] PPPM), followed by ICI + non-PBCT ($4648 [SD 24,323] PPPM).

Mean NSCLC-related healthcare costs during 1L treatment were $26,231 PPPM (SD 46,183) and were highest for ICI + PBCT ($31,690 [SD 44,358]). As with all-cause costs, most NSCLC-related costs were incurred in the outpatient setting ($23,388 [SD 40,111]), followed by inpatient costs ($2709 [SD 19,203]) (Supplementary Table S2).

The median and interquartile range for all-cause total costs by each treatment category are provided in Supplementary Table S3. Mean and median total healthcare costs were found to be similar.

## 4. Discussion

This study evaluated treatment patterns, HCRU and healthcare costs in the 1L treatment of metastatic NSCLC without actionable alterations in US real-world practice. While our study indicates widespread adoption of ICI-containing regimens in the 1L setting, it also identified a considerable burden in terms of HCRU and economic impact.

The most frequently used SACT in the 1L setting was ICI + PBCT (47%), followed by PBCT mono/combo (26%) and mono/dual ICI (19%). This is consistent with clinical practice guidelines, which recommend ICI + PBCT for 1L treatment [12,13]. Recent studies indicate a shift in treatment patterns compared with a 2018 study in which 93% received chemotherapy [17]. DaCosta Byfield and colleagues reported growing use of pembrolizumab (alone or in combination with chemotherapy) from fewer than 2% of claims in 2016 to almost half in 2018 (based on retrospective clinical data collected from a prior authorization tool linked with payer claims data from 2108 patients) [15]. Similarly, Kish and colleagues reported a decrease in the use of chemotherapy from 72% to 48% between 2017 and 2019, whereas the use of ICI + chemotherapy increased from 2% to 30% [26]. We found that 68% of regimens included an ICI (with or without chemotherapy), indicating that uptake has increased since earlier studies. It is possible that treatment patterns have changed within this data collection period, highlighting the importance of understanding how real-world treatment patterns evolve as new therapies are adopted into clinical use.

In the current study, treatment duration was found to be the lowest with chemotherapy-only regimens (2.7–4 months). This is consistent with the 2019–2021 US claims retrospective cohort study, which reported a shorter median time to treatment discontinuation with chemotherapy than with other regimens (2.4 months [IQR 2.1–3.7] vs. overall median of 3.4 [IQR 2.2–6.1]) [27], and a 2015–2018 study using claims data which reported shorter treatment duration with chemotherapy regimens compared with combined ICI + chemotherapy [15].

In the current study, all-cause outpatient, inpatient, and ED visits were all frequent with chemotherapy-only regimens, which is consistent with the greater toxicity of these regimens. Published studies have rarely compared HCRU associated with different treatment regimens in NSCLC. Pham and colleagues reported higher HCRU with ICI + chemotherapy combinations than with ICI alone [28], similar to the current study. A real-world study in Spain reported that HCRU for both disease management and management of adverse events was higher with chemotherapy than with ICIs; hospitalizations, ED visits and pharmacy visits were all more frequent with chemotherapy [29], as seen in the current study.

Outpatient visits were the main driver of HCRU in the 1L treatment of NSCLC in the current analysis, consistent with other studies [19,20,27]. However, the number of outpatient visits varies widely between studies. In the current study, the mean number of outpatient visits for all SACTs was 6.6 PPPM and ranged from 1.6 to 10.5 across the different treatment regimens. Spira and colleagues report a higher rate of mean outpatient visits (10.3 PPPM) in their study, which included fewer patients and appears to include targeted 1L treatments [27]. Notably, their study covered a different timeline from our study, which may also account for these differences. However, Spiral and colleagues reported similar numbers of inpatient stays (0.10 ± 0.3 PPPM) to the current study (0.12 PPPM) [27].

In terms of healthcare costs, the economic burden of non-targeted 1L treatment for metastatic NSCLC in the current study was $32,215 PPPM and was highest for patients receiving ICI in combination with chemotherapy ($34,721–38,454 PPPM). Consistent with the HCRU findings, the majority of healthcare costs were attributed to outpatient visits, potentially related to ICI-containing therapies. These costs are higher than those reported by Zhang and colleagues based on a US claims-based analysis of $22,633 PPPM, although this is an older study (claims from 2010 to 2019) and a largely commercially insured population [19]. Simmons and colleagues reported mean costs for 1L treatment of $36,717 ($35,658–37,777) PPPM for Medicare Advantage patients [20], which are similar to the costs reported in our study.

In the current study, total costs for 1L treatment were higher with ICI + chemotherapy regimens. Four other studies have reported a similar pattern. Kish and colleagues reported that total costs for 1L treatment were highest with ICI + chemotherapy ($32,436 PPPM) [26]. This study included a significant proportion of Medicaid patients (40%) who were not evaluated in our study, and the data were for a shorter and earlier period (January 2017 to May 2019). Spira and colleagues reported higher costs with pembrolizumab/immunotherapy plus chemotherapy compared with pembrolizumab/immunotherapy alone (US$44,678–45,682 vs. 31,774–33,371 PPPM) based on US claims data from January 2019 to June 2021 [27]. In another small claims-based study (2016–2021) of the 1L treatment of patients with advanced NSCLC and programmed death ligand 1 (PDL1) expression below 50% who made at least one claim for ICI-based chemotherapy, total adjusted all-cause medical costs were higher with ICI in combination than with chemotherapy alone (based on 88 patients in each group) [28]. The 2015–2018 study using claims data also identified higher total costs with pembrolizumab plus chemotherapy than with chemotherapy alone [15].

These studies together indicate that the 1L treatment of advanced/metastatic NSCLC without actionable alterations is associated with a substantial HCRU burden and healthcare costs. The existing literature suggests that newer therapies such as immunotherapies improve survival compared with chemotherapy alone [30,31] but are associated with higher upfront costs, as shown in the current analysis. While this study does not evaluate cost-effectiveness, our findings on the economic burden associated with 1L treatments provide valuable context for future studies evaluating the overall value of newer therapies. With the advent of novel therapies, real-world studies have become important to evaluate the value of treatment in terms of long-term and patient-reported outcomes and to guide evidence-based decision-making.

This study is subject to the limitations inherent in studies using administrative claims databases. There is no specific International Classification of Disease 10th edition (ICD10) code to define NSCLC, so patients with a lung cancer diagnosis code who were not receiving regimens for SCLC were determined to have NSCLC—an approach that has been used previously and validated against electronic medical record data [32]. History of smoking is based on claims related to nicotine dependence and is, therefore, likely to be underreported. An adaptation of a published algorithm was used to derive lines of treatment [23], which may differ from actual clinical practice. The Optum database used in this study mainly includes patients who are commercially insured or covered by Medicare Advantage plans; elderly patients with Medicare fee-for-service or Medicaid plans may be underrepresented, potentially limiting generalizability. Healthcare costs in this study are based on standardized costs reported in the database, which may not reflect actual reimbursements. Furthermore, this study descriptively assessed outcomes among patients with metastatic NSCLC and by 1L treatments received. Future studies could control the impact of patient characteristics to examine the association between 1L treatments and HCRU and costs.

## 5. Conclusions

The adoption of ICIs in the 1L treatment of metastatic NSCLC without actionable alterations has been increasing, and it is in line with treatment guidelines. However, a substantial economic burden persists, particularly with widely used ICI + chemotherapy regimens. Chemotherapy-only regimens accounted for a quarter of 1L treatments but were associated with the shortest treatment duration and high HCRU burden. The 1L treatment of metastatic NSCLC continues to impose a notable HCRU burden on patients, caregivers, and healthcare systems, as well as an economic burden for payers, highlighting the need for better treatment options in this setting.

## Figures and Tables

**Table 1:** Selection of study population.

Criterion	Number of Patients
Diagnosis of lung cancer (Dx date)	280,355
Diagnosis of secondary malignancy on or after Dx date	145,915
SACT for NSCLC on or after diagnosis of secondary malignancy	60,843
≥1 LOT initiated on or after 1 January 2020 (index date)	32,820
Continuously enrolled for ≥180 days before diagnosis to ≥30 days after index date	22,014
No medications are typically administered for SCLC before the index date	20,368
No claims for targeted treatment during the study period	19,055
No diagnosis of secondary malignancy or use of SACT within 180 days prior to Dx date	17,571
Age ≥ 18 years on the index date	17,571
1L SACT for NSCLC	15,659

Targeted therapies are those targeting actionable alterations; this does not include immunotherapies or therapies targeted at vascular endothelial growth factor (anti-angiogenic agents); Dx, diagnosis; LOT, line of treatment; 1L, first-line; NSCLC, non-small cell lung cancer; SACT, systemic anticancer treatment; SCLC, small cell lung cancer.

**Table 2:** Baseline characteristics (*n* = 15,659).

Characteristics		Value
Age, years, mean (SD)		71.7 (8.6)
Sex, male, %		51.8
US census region, %	Northeast	14.1
	Midwest	26.1
	South	43.4
	West	16.2
Medicare Advantage, %		85.5
Index year, %	2020	30.3
	2021	30.2
	2022	33.7
	2023 Q1	7.7
Comorbidities, %	Hypertension	72.7
	COPD	44.3
	Coronary heart disease	29.2
	Anemia	28.8
	Diabetes	28.5
	Chronic kidney disease	14.8
	Brain/spinal cord	14.1
	Chronic liver disease	12.5
	Dementia	1.7
	Cerebrovascular disease	1.1
	Other	64.2
NCI-CCI, mean (SD)		2.3 (1.9)
Secondary metastases, %	Bone	21.6
	Brain/spinal cord	14.1
	Liver	11.3
	Other	64.2
History of smoking, % ^a^		79.8
Time from lung cancer diagnosis, months, median (IQR)		1.5 (0.9–2.8)

^a^ History of smoking is based on claims related to nicotine dependence and is, therefore, likely to be underreported. COPD, chronic obstructive pulmonary disease; IQR, interquartile range; NCI-CCI, National Cancer Institute-Adapted Charlson Comorbidity Index; Q, quarter; SD, standard deviation.

## Data Availability

The datasets used in this study were obtained from a third-party source and are subject to data-sharing restrictions. Consequently, the datasets generated and analyzed during this study are confidential and cannot be shared by the authors or Amgen.

## Supplementary Materials

The following supporting information can be downloaded at https://www.mdpi.com/article/10.3390/curroncol32030151/s1, Supplementary Table S1. All-cause and NSCLC-related healthcare resource utilization during first-line treatment; Supplementary Table S2. All-cause and NSCLC-related healthcare costs during first-line treatment. Supplementary Table S3. Median all-cause healthcare costs ($, per patient per month) during first-line treatment.

## Abbreviations

The following abbreviations are used in this manuscript:

1L	first-line
2L	second line
COPD	chronic obstructive pulmonary disease
ED	emergency department
HCRU	healthcare resource utilization
ICD-10-CM	The International Classification of Diseases, 10th Edition, Clinical Modification
ICI	immune checkpoint inhibitor
KRAS	Kirstin rat sarcoma virus
NCI-CCI	National Cancer Institute-adapted Charlson Comorbidity Index
NCCN	National Comprehensive Cancer Network
NSCLC	non-small cell lung cancer
PBCT	platinum-based chemotherapy
PPPM	per patient per month
SACT	systemic anticancer therapy
SD	standard deviation
STROBE	Strengthening the Reporting of Observational Studies in Epidemiology
TTNT	time to the next treatment
