# Supplementary material for: Real-World Treatment Patterns, Healthcare Resource Utilization, and Healthcare Costs in the First-Line Treatment of Metastatic Non-Small Cell Lung Cancer in the US
Source: Curr Oncol. 2025 Mar 5;32(3):151. doi: 10.3390/curroncol32030151 (PMC11940980; doi:10.3390/curroncol32030151)
Supplement: Supplementary file 1 [file curroncol-32-00151-s001.zip › curroncol-3459058-supplementary.pdf]

Real-World Treatment Patterns, Healthcare Resource Utilization, and Healthcare Costs in the First-Line Treatment of Metastatic Non-Small Cell Lung Cancer in the US

Supplementary Table S1. All-cause and NSCLC-related healthcare resource utilization during first-line treatment

HCRU, PPPM	All	ICI + PBCT	ICI + non-PBCT	ICI mono / dual	PBCT mono / combo	Non-PBCT mono	Non-PBCT combo	Other SACT
n	15,659	7359	176	3089	4139	437	181	278
**All-cause**								
Prescriptions	3.93	4.05	3.79	3.33	4.32	4.32	4.28	3.36
ED visits	0.11	0.11	0.13	0.10	0.16	0.12	0.13	0.11
Outpatient visits	6.56	6.37	5.77	4.65	10.54	6.64	6.23	4.62
Physician office	3.00	2.96	2.84	2.22	4.38	3.16	3.05	2.73
Hospital outpatient	3.56	3.41	2.94	2.43	6.16	3.48	3.17	1.89
Inpatient admissions	0.12	0.12	0.15	0.11	0.16	0.16	0.19	0.07
Hospitalizations	0.11	0.11	0.14	0.10	0.15	0.15	0.17	0.06
Other	0.01	0.01	0.02	0.01	0.01	0.01	0.02	0.01
ICU admissions	0.05	0.05	0.06	0.04	0.07	0.07	0.07	0.03
Inpatient days	0.95	0.87	1.25	0.87	1.32	1.30	1.40	0.64
Hospital	0.80	0.75	1.06	0.70	1.13	1.04	1.12	0.49
Other	0.15	0.12	0.19	0.17	0.19	0.26	0.29	0.15
**NSCLC-related**								
Prescriptions	0.01	0.01	0.03	0.01	0.02	0.01	0.05	0.00
ED visits	0.06	0.06	0.05	0.04	0.09	0.02	0.03	0.00
Outpatient visits	4.86	5.19	2.85	2.40	8.53	1.33	0.95	0.43
Physician office	2.05	2.25	1.23	1.01	3.36	0.55	0.44	0.20
Hospital outpatient	2.81	2.94	1.62	1.39	5.17	0.78	0.51	0.23

Inpatient admissions	0.10	0.10	0.10	0.07	0.13	0.04	0.04	0.01
Hospitalizations	0.09	0.10	0.09	0.06	0.12	0.04	0.04	0.01
Other	0.01	0.01	0.01	0.01	0.01	0.00	0.00	0.00
ICU admissions	0.04	0.04	0.03	0.03	0.06	0.02	0.02	0.00
Inpatient days	0.76	0.78	0.82	0.57	1.07	0.36	0.29	0.06
Hospital	0.65	0.68	0.69	0.47	0.93	0.31	0.26	0.04
Other	0.10	0.10	0.14	0.10	0.14	0.04	0.03	0.02

ED, Emergency Department, HCRU, healthcare resource utilization; ICI, Immune checkpoint inhibitor; ICU, intensive care unit; NSCLC, non-small cell lung cancer; PBCT, Platinum-based chemotherapy; PPPM, per patient per month; SACT, systemic anticancer therapy

Supplementary Table S2. All-cause and NSCLC-related healthcare costs during first-line treatment

Healthcare costs, $, PPPM	All (n=15,659)		ICI + PBCT (n=7,359)		ICI + non-PBCT (n=176)		ICI mono / dual (n=3,089)		PBCT mono / combo (n=4,139)		Non-PBCT mono (n=437)		Non-PBCT combo (n=181)		Other (n=278)	
	Mean	SD	Mean	SD	Mean	SD	Mean	SD	Mean	SD	Mean	SD	Mean	SD	Mean	SD
All-cause																
Total	32,215	44,597	34,721	44,191	38,454	55,678	31,340	46,696	26,271	41,359	20,245	32,251	25,039	38,762	13,957	29,946
Prescriptions	658	4,919	523	4,195	611	5,094	1,062	7,805	537	2,603	898	4,745	948	3,800	982	6,561
Medical	31,557	44,481	34,198	44,153	37,843	55,904	30,277	46,118	25,734	41,294	19,346	32,194	24,091	38,972	12,975	28,975
ED visits	100	2,966	114	3,777	282	10,598	66	1,722	75	525	95	1,981	73	685	125	1,833
Outpatient	28,045	37,735	30,888	37,409	32,913	50,790	27,185	38,529	20,933	33,654	14,848	22,759	19,446	33,717	10,723	21,492
Outpatient visits	27,187	36,781	30,051	36,384	31,918	50,815	26,398	37,209	20,006	32,969	13,692	22,272	17,286	26,108	9,899	20,672
Physician office visits	8,829	28,867	10,180	35,016	11,066	43,416	8,637	28,850	4,561	11,501	4,373	15,641	4,991	16,697	3,729	11,641
Hospital outpatient visits	18,358	43,889	19,871	48,378	20,852	56,741	17,761	45,144	15,445	35,072	9,319	20,221	12,294	27,369	6,170	21,097
Other outpatient	858	8,496	837	8,964	995	4,204	787	8,484	927	6,869	1,156	5,737	2,160	23,299	824	4,142
Inpatient	3,412	21,791	3,196	20,683	4,648	24,323	3,027	22,944	4,726	22,874	4,404	21,888	4,572	17,064	2,127	19,041
Hospital	3,263	20,953	3,079	20,116	4,429	23,785	2,838	21,893	4,520	21,729	4,173	21,164	4,344	15,942	2,001	18,406
Other inpatient	149	4,094	116	2,213	219	2,382	189	4,944	206	5,906	230	2,452	229	3,455	127	1,582
NSCLC-related																
Total	26,231	46,183	31,690	44,358	21,519	58,484	18,842	44,454	21,633	42,300	4,439	20,758	4,459	18,229	1,081	6,266
Prescriptions	66	2,251	65	2,517	199	4,838	85	2,693	28	910	71	1,761	189	2,680	14	557
Medical	26,165	46,230	31,626	44,458	21,321	58,522	18,757	44,421	21,606	42,319	4,369	20,732	4,271	18,211	1,067	6,252
ED visits	68	2,575	94	3,697	19	148	25	838	48	493	11	127	15	126	2	39
Outpatient	23,388	40,111	28,683	38,344	18,408	50,896	16,823	38,890	17,679	34,805	3,151	14,465	3,288	15,190	888	5,336
Outpatient visits	22,837	39,455	28,071	37,874	17,912	50,192	16,431	38,130	17,021	33,966	2,948	14,030	3,087	14,775	852	5,203
Physician office visits	7,466	27,376	9,591	34,364	4,500	22,822	5,488	24,273	3,799	10,790	917	7,146	846	6,311	257	2,593
Hospital outpatient visits	15,371	42,451	18,480	47,570	13,412	51,010	10,942	37,705	13,223	35,019	2,032	11,964	2,242	13,472	595	4,498
Other outpatient	551	5,513	612	5,760	496	2,544	392	4,041	658	6,552	202	1,432	201	1,100	36	388
Inpatient	2,709	19,023	2,848	19,063	2,895	21,939	1,909	16,847	3,878	21,538	1,207	11,904	968	5,989	177	2,586
Hospital	2,603	18,331	2,752	18,585	2,750	21,472	1,802	16,099	3,715	20,482	1,170	11,236	936	5,852	164	2,566
Other inpatient	106	3,671	97	2,036	144	2,028	107	3,602	163	5,792	37	1,245	32	677	13	352

ED, Emergency Department, HCC, healthcare costs; ICI, Immune checkpoint inhibitor; NSCLC, non-small cell lung cancer; PBCT, Platinum-based chemotherapy; PPPM, per patient per month; SACT, Systemic anticancer therapy

Supplementary Table S3. Median all-cause healthcare costs ($, per patient per month) during first-line treatment

All (n=15,659)		ICI + PBCT (n=7,359)		ICI + non-PBCT (n=176)		ICI mono / dual (n=3,089)		PBCT mono / combo (n=4,139)		Non-PBCT mono (n=437)		Non-PBCT combo (n=181)		Other (n=278)	
**Median**	**IQR**	**Median**	**IQR**	**Median**	**IQR**	**Median**	**IQR**	**Median**	**IQR**	**Median**	**IQR**	**Median**	**IQR**	**Median**	**IQR**
29,119	19,064	32,103	19,169	33,946	26,536	26,813	13,545	21,298	20,867	15,771	20,131	20,021	17,877	10,729	13,559

ICI, immune checkpoint inhibitor; IQR, interquartile range; PBCT, platinum-based chemotherapy
